# Orbitofrontal functional network: the mediating role between violence and childhood trauma in patients with schizophrenia

**DOI:** 10.1038/s41537-025-00666-2

**Published:** 2025-10-07

**Authors:** Juntao Lu, Ningzhi Gou, Qiaoling Sun, Ying Huang, Huijuan Guo, Jingyan Sun, Jiansong Zhou, Xiaoping Wang

**Affiliations:** 1 Department of Psychiatry, National Clinical Research Center for Mental Disorders, and National Center for Mental Disorders, The Second Xiangya Hospital of Central South University, Changsha, Hunan China; 2 Department of Psychiatry, the First Affiliated Hospital, Medical College of Xi ‘an Jiaotong University, Xi’an, Shaanxi China

**Keywords:** Biomarkers, Schizophrenia

## Abstract

A growing body of evidence has indicated an increased risk of violence among patients with schizophrenia. While childhood trauma (CT) has been robustly associated with increased violent behavior in schizophrenia spectrum disorders, the neurobiological mechanisms underlying this relationship remain underexplored. The objectives of this study are to investigate the potential role of functional connectivity (FC) in the relationship between CT and violence. This study enrolled 55 patients with schizophrenia and 36 healthy controls. Seed-based functional connectivity between a predefined seed in the orbitofrontal cortex and other brain voxels was compared across groups, with significant results regarding FC used in further mediation analysis. The seed-based analysis revealed decreased FC between the right orbital part of the inferior frontal gyrus (ORBinf) and the right middle temporal gyrus as well as the right superior frontal gyrus in violent schizophrenia patients (VSP) compared to both healthy controls (HC) and non-violent schizophrenia patients (NVSP). VSP also exhibited decreased FC between the right ORBinf and the right middle frontal gyrus compared to NVSP. Furthermore, the mediation analysis indicated that the relationship between CT and violence was completely mediated by the strength of FC between the right ORBinf and the right MTG. The present study suggested that alterations of FC between certain brain regions may be associated with violence and offer valuable insights into potential neural targets for interventions aiming to address CT and violence in patients with schizophrenia.

## Introduction

Numerous studies have revealed an association between violence and schizophrenia^1,2^. Although this relationship is often overstated, it has been acknowledged that patients with schizophrenia spectrum disorders are at an increased risk of violence, especially homicide, compared to healthy individuals^3,4^, with a study suggesting that patients with schizophrenia have an 18-fold increased risk of violence than those without schizophrenia^1^. Thus, a better understanding of violence in this patient population may be of great significance for preventing potential harm and reducing stigma. Previous studies have found that some clinical factors, such as hallucination, delusion^5^, and impulse control as well as some socio-demographic factors such as childhood trauma, age, and history of violence^6,7^ might be contributive to the violence in schizophrenia.

Childhood trauma (CT) is one of the risk factors associated with severe violence such as murder in patients with schizophrenia^7^. Patients with early-onset psychosis who have experienced child abuse are found to be more likely to exhibit violent behaviors later in life^8^. A meta-analysis revealed that patients with psychosis who had a history of CT were at a 2-fold increased risk of violence compared to those without such a history^9^. The association between exposure to childhood physical abuse and later perpetration of violent behavior has also been found in the general population^10,11^. Despite the previous findings that exposure to CT is more prevalent among patients with schizophrenia^12^ and that this exposure may increase the risk of violence, the neural mechanism underlying the association between CT and violence remains unclear in schizophrenia.

In recent years, neuroimaging studies have found the orbitofrontal cortex (OFC) may be a neural basis for the association between CT and individuals^13,14^. For instance, a study found that patients with mental disorders who had a history of CT demonstrated decreased connectivity between bilateral OFC and left nucleus accumbens, as compared to those without the exposure^15^. A recent study found a reduction in gray matter volume in the right medial orbitofrontal region among both healthy individuals and patients with mental disorders who were exposed to CT^16^. Furthermore, the interaction between CT, recent stressful life events, and orbitofrontal cortex structure was found in a large-scale study^17^, indicating that exposure to CT might affect both the structure and the function of OFC.

Interestingly, the OFC has also been implicated in violent behavior. Although neuroimaging studies on violence in patients with schizophrenia are heterogeneous, alterations in the OFC are among the most consistent findings in violent patients with schizophrenia^18^. The OFC is a key region implicated in impulse control; studies indicated that the structural deficit of OFC was associated with impulsivity, and that OFC might be involved in the neural circuitry of impulsivity and aggression^19,20^. Functional studies also found that the connectivity between the OFC and other brain regions was reduced in patients with schizophrenia, and the strength of this connectivity was negatively associated with the level of aggression as reflected by the Buss-Perry Aggression Questionnaire^21^. Athanassiou et al. found that the functional connectivity between the OFC and the anterior cingulate cortex was associated with violent behavior in schizophrenia and that the connectivity was predictive of the number of violent behaviors^22^. Diminished activation of the OFC in response to anger has also been found in psychiatric patients with aggressive behavior compared to healthy controls (HC), and the patients failed to demonstrate coupling of the OFC with other brain regions compared to controls^23^, which provided additional support for the abnormal activity and connectivity of the OFC in violent patients. Taken together, current findings have provided empirical evidence that exposure to CT might affect the function of specific brain regions such as the OFC, which is believed to be associated with violent behavior.

Despite the numerous studies on the association between violence and CT in patients with schizophrenia, only one study investigated the relationship between CT and aggression from a neural perspective among healthy individuals^24^. To our knowledge, there is a lack of research linking CT-related brain regions to violent behaviors in schizophrenia. In addition, the majority of studies primarily focused on adolescents, which limited our understanding of how the neurobiological consequences of CT contributed to violence in adulthood. Therefore, it is imperative to explore the underlying neural mechanisms of CT and violence in schizophrenia.

The objective of our study was to explore the resting-state FC network of OFC whether associated with violence, and to investigate the potential role of FC strength in the relationship between CT and violence. We hypothesized that patients with schizophrenia who had a history of violence might exhibit abnormal FC of OFC compared to those without the history and healthy controls, and that the FC strength of OFC might mediate the relationship between violence and CT in patients with schizophrenia.

## Methods

### Participants

A total of 91 male participants were enrolled in this study, including 55 patients with schizophrenia recruited from the Second Xiangya Hospital (Changsha, Hunan, China) and 36 HC recruited from the community. The patients were divided into the violent schizophrenia patient (VSP) group (consisting of 22 patients) and the non-violent schizophrenia patient (NVSP) group (consisting of 33 patients) based on their history of violent behavior. Violence was defined as behaviors causing severe physical injuries to others within one month, including homicide and other serious assaults. All VSP participants had engaged in such violent behaviors and were referred to the forensic psychiatry department of the Second Xiangya Hospital for examination; for a better quantitative description, patients assigned to the VSP group should also score over 4 on the Modified Overt Aggression Scale (MOAS)^25^. The inclusion criteria for this study were 1) patients who met the criteria for schizophrenia according to the International Classification of Diseases Version 10 (ICD-10); 2) participants who were aged between 18 and 65 years; 3) participants who had completed at least 6 years of education. Participants who met the following criteria were excluded: 1) having MRI contraindications, 2) with a history of severe head injury that caused loss of consciousness, and 3) being diagnosed with other psychiatric disorders, including had a history of substance abuse or dependence.

Prior to enrollment, each participant received comprehensive details about the research protocol and subsequently signed an informed consent document. The study was approved by the Ethics Board of the Second Xiangya Hospital of Central South University and conforms to the provisions of the Declaration of Helsinki.

### Collection of socio-demographic and clinical data

The socio-demographic and clinical information was collected using a self-designed standardized form, which included age, level of education, age of onset, duration of untreated psychosis (DUP), and duration of disease. The dose equivalents of antipsychotics were calculated based on defined daily doses (DDDs)^26^.

Childhood maltreatment was assessed using the Childhood Trauma Questionnaire (CTQ)^27^, a reliable and valid self-administered inventory that retrospectively assesses CT. This 28-item version assesses five aspects of CT, i.e., physical abuse, emotional abuse, sexual abuse, physical neglect, and emotional neglect^28^.

Positive and Negative Syndrome Scale (PANSS)^29^ was used to assess the symptoms of the patients with schizophrenia. With good internal-consistency reliability, the PANSS assesses the patients from five perspectives, i.e., disorganization, excitement, depression, positive symptoms, and negative symptoms^30^.

Violent behavior within the past month was assessed using the MOAS^31^, from four aspects: verbal aggression, aggression towards objects, auto-aggression, and physical aggression. This scale has proven to have good validity in the Chinese population^32^.

### Acquisition and processing of MRI data

A 3 T Philips scanner was used to acquire whole-brain functional MRI data from all participants with the following parameters: matrix = 64 × 64, field of view (FOV) = 240 × 240 mm^2^, flip angle (TR) = 2000 ms, echo time (TE) = 30 ms, flip angle = 90°, slices thickness = 4 mm, voxel size = 3 × 3 × 3 mm^3^, and 240 volumes.

Image processing and all analyses were conducted on the Matlab R2021a platform. The Statistical Parametric Mapping version 12 (SPM12 https://www.fil.ion.ucl.ac.uk/spm/software/spm12/) and Data Processing & Analysis for (Resting-state) Brain Imaging (DPABI, http://rfmri.org/dpabi)^33^ were used to preprocess the functional data. First, the first 10 images were removed to avoid potential noise during participant adaptation. The data were then corrected for acquisition delay between slices. Then, subjects with mean frame-wise displacement (FD) greater than 0.4 mm were excluded after realignment. The images were normalized to standard Montreal Neurological Institute (MNI) space and resampled to 3*3*3 m^3^ using the tool of Diffeomorphic Anatomical Registration using Exponentiated Lie algebra (DARTEL)^34^. Then, nuisance signals, including Friston-24 head motion parameters, white matter signals, and cerebrospinal fluid signals, were regressed out, and the functional images were smoothed using a 6-mm full-width at half maximum (FWHM). Lastly, the impact of low-frequency drifts was reduced by band-pass filtering at 0.01–0.08 Hz.

### Seed-based functional connectivity analyses

The DPABI was used to analyze seed-based FC. To investigate the role of the orbit frontal cortex (OFC) in the association between childhood trauma and violence, We defined seed regions based on the Automated Anatomical Labeling (AAL) template within the orbitofrontal cortex using the MNI coordinates of its three anatomical subdivisions: the orbital parts of the superior, middle, and inferior frontal gyri (sphere radius = 3 mm)^35^. The correlation of the mean blood-oxygen-level-dependent time series was calculated for each pair of seed regions of interest (ROIs) and all the other voxels in the whole brain. The FC maps calculated in the previous step were converted with Fisher’s Z transformation to improve normality.

### Statistical analyses

Normality of all scales was evaluated both visually and via the Shapiro–Wilk test. One-way analysis of variance (ANOVA) was used for inter-group comparison of continuous variables, including age and years of education, and independent samples t test was used to compare continuous clinical variables between VSP and NVSP. For non-normally distributed data, non-parametric tests were applied (Kruskal–Wallis test for multiple groups; Mann–Whitney U test for two groups). Chi-square (χ2) test was used for the analyses of categorical variables. All the above analyses were performed using Statistical Package for Social Sciences Version 25.0, with the level of significance set at *P* = 0.05 (two-tailed).

The zFC map was analyzed using one-way ANOVA between the VSP, NVSP and HC groups with the level of education and age as covariates. The significant ROIs in ANOVA were extracted as masks for post-hoc t test between each two groups. The Gaussian random field (GRF) was applied for the correction of multiple comparison analyses to control fake positive results, with a voxel-wise *P* < 0.001 and a cluster-wise *P* < 0.05 (two-tailed).

The mediation analysis was conducted using AMOS 28 to analyze the process and mechanism linking childhood maltreatment to violence. Considering significant effects of childhood trauma on the strength of FC, a mediating effect model was employed to further analyze the relationship between childhood trauma and violence. Mediation analyses were conducted only for CTQ subscales with significant group differences between VSP and NVSP to focus on trauma dimensions most relevant to violent behavior in schizophrenia.

## Results

### Demographic and clinical variables

**Fig. 1:**
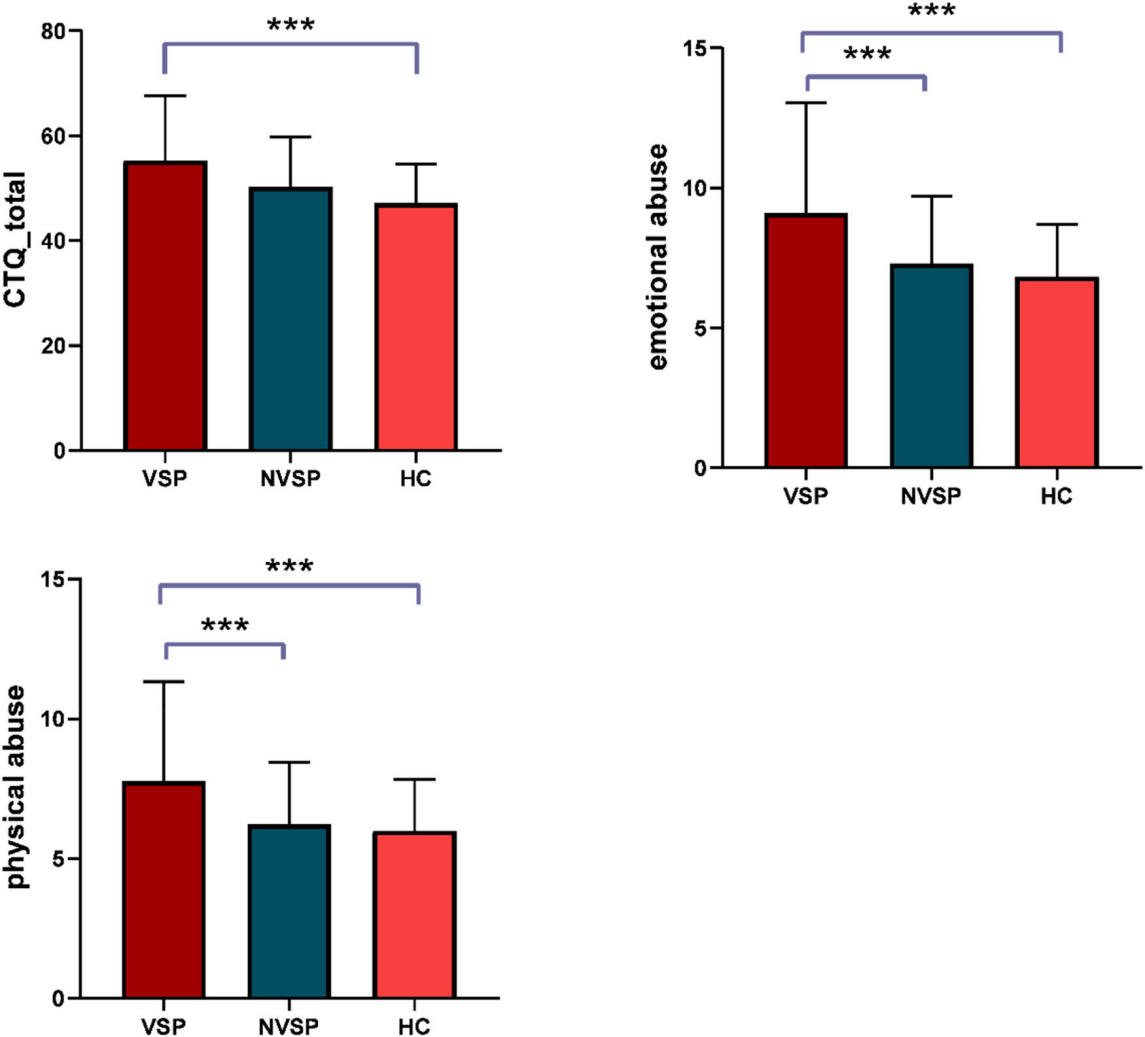
Comparison of scores of CTQ and its subscales between VSP, NVSP, and HC.

The demographic, clinical and CTQ data are presented in Table 1. No significant differences were found between the three groups in terms of age, while HC had a significantly higher level of education than the two patient groups. The DUP, age of onset, duration of illness, positive symptoms, DDDs, and disorganization were not significantly different between the VSP and NVSP groups. As expected, VSP had significantly higher scores of excitement (in PANSS) and MOAS than NVSP. In addition, the three groups showed significant difference s in the total score of CTQ as well as scores of emotional abuse and physical abuse. Post hoc t test revealed significantly higher total score and scores of emotional abuse and physical abuse in CTQ in VSP than in HC, as well as significantly higher scores for physical abuse and emotional abuse in VSP than in NVSP (Fig. 1). It is worth noting that no significant difference was found between NVSP and HC in all aspects of CTQ.

**Table 1:** Demographic and clinical information of HC, NVSP, and VSP.

Characteristics	VSP (*n* = 22)	Nvsp (*n* = 33)	Hc (*n* = 36)	Statistics		Post hoc tests	
	Mean (SD)	Mean (SD)	Mean (SD)	F/t	*p*		
Age	30.0 ± 7.6	31.2 ± 8.0	30.9 ± 6.7	0.207	0.813		
Sex (M/F)	22/0	33/0	36/0	-	-		
Education level (years)	10.3 ± 2.5	12.8 ± 3.9	14.4 ± 2.7	11.517	<0.001		
Age of onset	23.0 ± 7.6	24.7 ± 8.0	NA	−0.771	0.444		
DUP (months)	18.1 ± 27.1	23.3 ± 36.2	NA	−0.529	0.599		
Duration of illness	69.6 ± 62.1	82.1 ± 70.8	NA	−0.682	0.499		
DDDs	9.17 ± 7.95	11.58 ± 7.81	NA	−1.113	0.271		
PANSS							
Positive symptoms	13.4 ±± 3.5	13.0 ± 3.6	NA	0.388	0.699		
Negative symptoms	15.6 ± 4.6	11.0 ± 4.0	NA	3.897	<0.001		
Disorganization	5.6 ± 2.5	5.5 ± 2.4	NA	0.114	0.910		
Excitement	12.5 ± 5.1	6.8 ± 2.2	NA	4.949	<0.001		
Depression	3.9 ± 1.8	4.2 ± 2.1	NA	-0.445	0.658		
MOAS	10.9 ± 7.7	0.24 ± 1.2	NA	6.427	<0.001		
CTQ total score	55.3 ± 12.4	50.3 ± 9.5	47.1 ± 7.5	4.493	0.014	VSP > HC	
Emotional abuse	9.1 ± 3.9	7.3 ± 2.4	6.8 ± 1.9	4.646	0.012	VSP > HC	VSP>NVSP
Physical abuse	7.8 ± 3.6	6.2 ± 2.2	6.0 ± 1.8	3.464	0.036	VSP > HC	VSP> NVSP
Sexual abuse	6.2 ± 1.7	6.4 ± 2.6	5.9 ± 1.6	0.662	0.519	NA	
Emotional neglect	13.6 ± 4.7	12.2 ± 4.8	11.8 ± 4.8	3.021	0.054	NA	
Physical neglect	10.9 ± 3.7	9.7 ± 3.2	9.0 ± 3.0	2.035	0.137	NA	

*DUP* duration of untreated psychosis, *PANSS* Positive and Negative Syndrome Scale, *MOAS* Modified Overt Aggression Scale, *CTQ* Childhood Trauma Questionnaire, *DDDs* defined daily doses.

### Seed-based functional connectivity

**Fig. 2:**
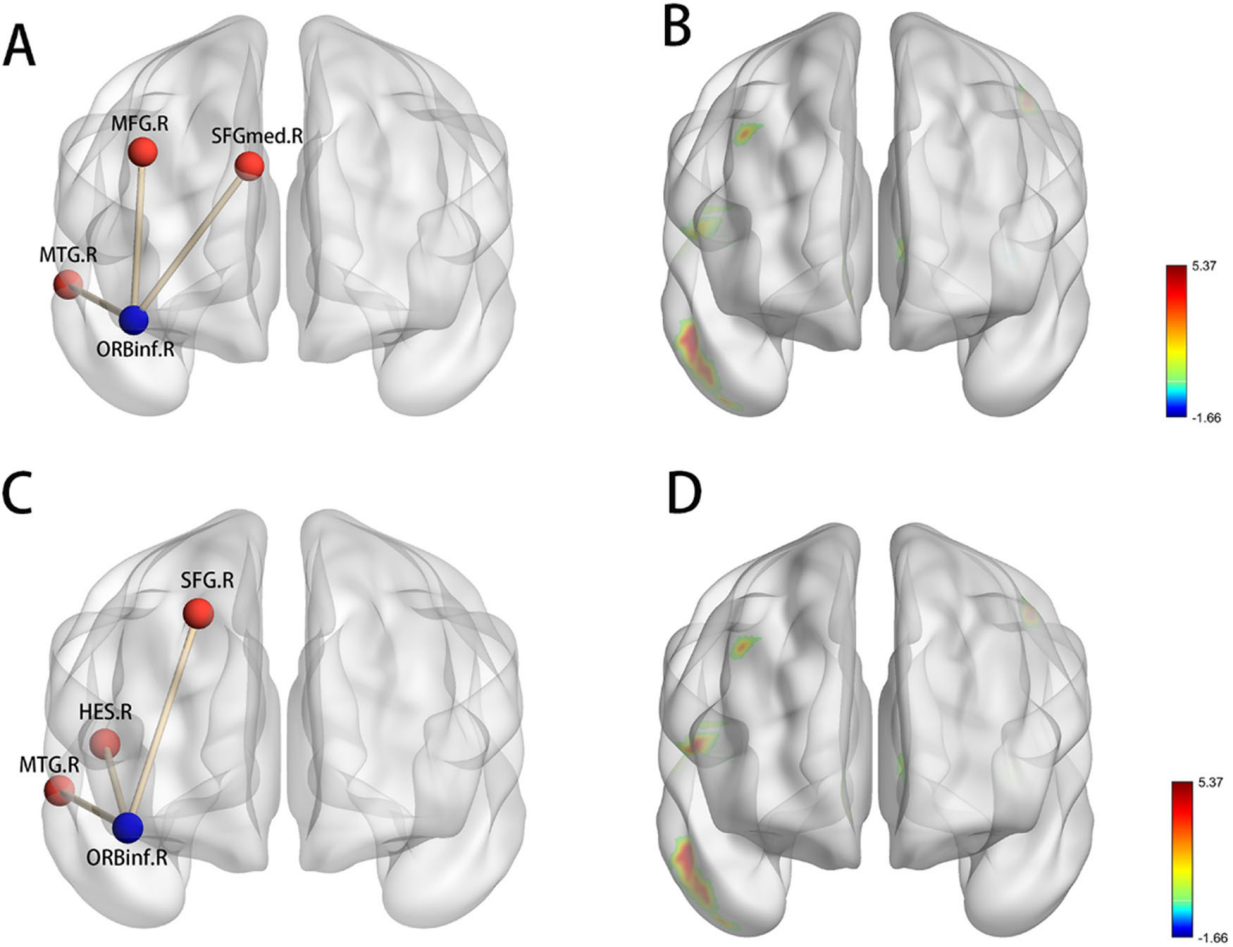
Decreased functional connectivity in VSP compared to NVSP and HC. **A**, **B** Decreased FC in VSP compared to NVSP (ORBinf the orbital part of the inferior frontal gyrus, MTG middle temporal gyrus, MFG middle frontal gyrus, SFGmed superior frontal gyrus, medial), **C**, **D** Decreased FC in VSP compared to HC (ORBinf the orbital part of the inferior frontal gyrus, MTG middle frontal gyrus, HES Heschl gyrus, SFG superior frontal gyrus).

According to the ANOVA, the HC, NVSP, and VSP groups exhibited different FC in the right orbital part of the inferior frontal gyrus (ORBinf) and right middle temporal gyrus (MTG), the right orbital part of the superior frontal gyrus (ORBsup), the right rolandic operculum (ROL), and the right middle frontal gyrus (MFG). Post hoc t test showed decreased FC between the right ORBinf and the right MTG as well as the right MFG and the right superior frontal gyrus, medial (SFGmed) in VSP compared with NVSP (Fig. 2A, B). VSP also exhibited decreased FC between the right ORBinf and other brain regions, including the right MTG, the right superior frontal gyrus (SFG), and the right Heschl gyrus (HES), as compared to HC (Fig. 2C, D). No significant difference was found in the post hoc t test between the HC and NVSP groups. All the above results passed the multiple comparison analysis (voxel level, *P* < 0.001, cluster level, *P* < 0.05, GRF corrected) (Table 2).

**Table 2:** Functional connectivity alterations in HC, NVSP and VSP.

	Regions	L/R	Cluster size	F/T value	MNI coordinates
ANOVA	Middle temporal gyrus	R	51	14.39	51 −3 −21
	Orbital part of the superior frontal gyrus	R	16	10.75	18 54 −3
	Rolandic operculum	R	30	12.28	51 −6 9
	Middle frontal gyrus	R	12	12.29	33 24 36
nvsp > vsp	Middle temporal gyrus	R	47	4.81	54 0 −21
	Superior frontal gyrus, medial	R	16	5.37	15 51 0
	Middle frontal gyrus	R	12	5.20	33 24 36
hc > vsp	Middle temporal gyrus	R	42	4.53	54 −3 −18
	Superior frontal gyrus	R	16	5.18	18 54 0
	Heschl gyrus	R	29	5.38	54 -6 6

GRF corrected with voxel level *P* < 0.001; cluster significance *P* < 0.05, two-tailed.

*L* left, *R* right, *MNI* Montreal Neurological Institute.

### Mediation analysis

**Fig. 3:**
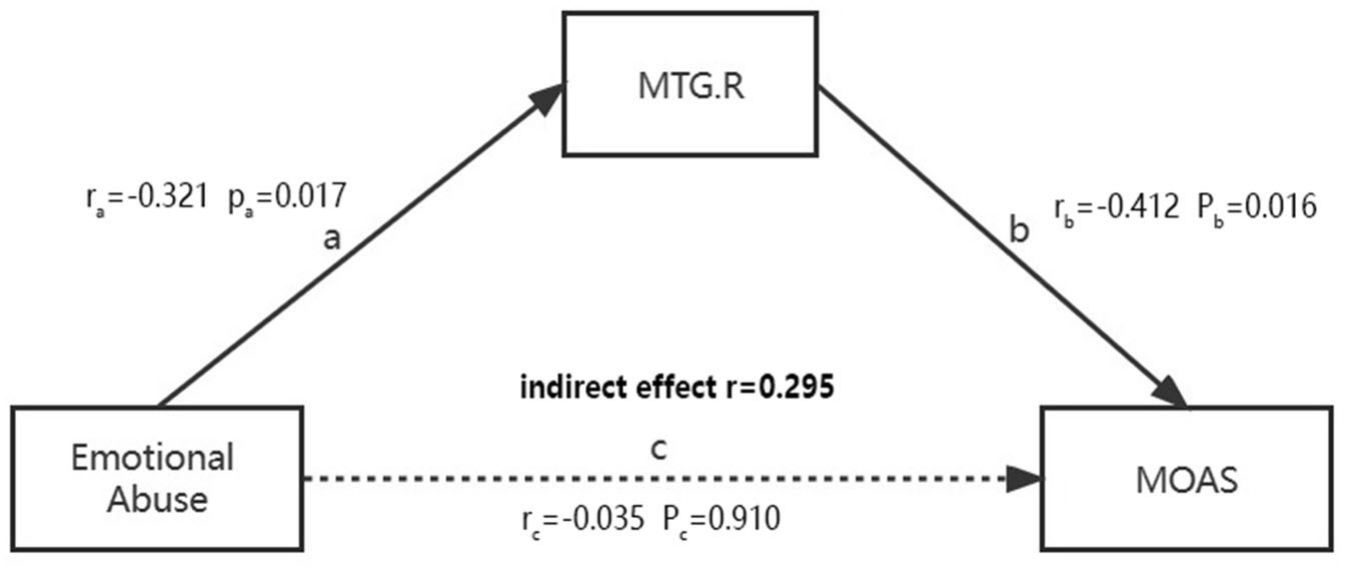
The mediation effect of the FC strength between childhood trauma and violence in patients with schizophrenia.

The zero-order correlations showed emotional abuse was significantly negatively correlated with FC between the right ORBinf and the right MTG (*r* = −0.321, *p* = 0.026), and FC was significantly negatively correlated with MOAS scores (*r* = −0.423, *p* = 0.003). However, there was no significant direct correlation between emotional abuse and MOAS scores (*p* = 0.255). The mediation analysis showed that the strength of FC between the right ORBinf and the right MTG was negatively correlated with scores of emotional abuse (Fig. 3, *r*a = −0.321) and MOAS (Fig. 3, *r*b = −0.412) in patients with schizophrenia. The model fit indices indicated a good fit, with RMSEA = 0.005 and CFI = 1.000. The bootstrap 95% confidence interval for the indirect effect was [0.070, 0.678], which did not include zero, suggesting a significant mediation pathway. In contrast, the bootstrap 95% confidence interval for the direct effect was [−0.705, 0.746], indicating a non-significant direct effect. It was also found that the relationship between emotional abuse and MOAS was mediated by the FC between ORBinf and MTG (indirect effect *r* = 0.295). Thus, the strength of FC played a complete mediating role in regulating the relationship between childhood trauma and violence in this sample.

## Discussion

Several significant and valuable results were found in the present study. Firstly, individuals in the VSP group experienced more severe physical and emotional abuse compared to both the NVSP and HC groups, which highlighted the significant role of CT in contributing to violent behavior among patients with schizophrenia. Secondly, VSP exhibited decreased FC of the right ORBinf with the right MTG and the right SFG compared to HC and NVSP; VSP also exhibited decreased FC between the right ORBinf and the right MFG compared to NVSP, as well as between the right ORBinf and the right HES compared to HC. Additionally, this study indicated that the FC might regulate the relationship between CT and violence in patients with schizophrenia. Notably, the relationship between CT and violence was completely mediated by the FC between the right ORBinf and the right MTG. Taken together, the present study may provide evidence for the role of CT in the risk of violence and the role of FC networks in the association between violence and CT.

The post hoc t test also revealed that VSP had experienced more severe CT than HC and NVSP, while no significant difference was found between NVSP and HC in this regard. Despite numerous studies indicating that patients with schizophrenia are more likely to report exposure to CT than controls^36,37^, most of those studies analyzed violent and non-violent patients as one group, which precluded them from looking into the impact of violence on the relationship between CT and schizophrenia. The association between childhood neglect and violence has also attracted increasing attention over the past years^38,39^. Sun et al. found that exposure to CT could increase the risk of violence in later life in patients with schizophrenia, which supports our findings^40^. A cohort study found patients with CT may showed pervasive cognitive deficits, include impaired executive function which often correlated with violence^41,42^. A study found that patients with schizophrenia who had a history of CT often perceived negative faces as more negative and positive faces as less positive^43^, and a study also indicated that abnormal perception of angry faces might be a characteristic of violent patients with schizophrenia^44^. In addition, altered structure and connectivity of brain regions such as the limbic system and the frontal lobe have been found in individuals with CT^45,46^, and these alterations were also found to be associated with violence in schizophrenia^47^

Decreased FC between ORB and frontotemporal regions was found in VSP compared to HC and NVSP, however, the FC network of ORB showed no significant difference between NVSP and HC, suggesting that the altered ORB network might be associated with violence rather than disease itself. Consistent with our results, abnormal activation of frontal and temporal regions was found in violent patients with schizophrenia compared to non-violent patients when they were exposed to sustained visual threat cues^48^. The MTG and the HES are important parts of the temporal lobe. Previous studies suggest that abnormalities in these areas may be related to impaired aggression control, increased impulsivity, and emotional processing deficits^49^. Studies have also found that the MTG and the HES are associated with auditory and language processing, with connections to other pivotal brain regions involved in language pathways^50,51^. The FC between MTG or HES and other brain regions was found to be associated with auditory verbal hallucinations in patients with schizophrenia^52,53^, which may interpret the difference between VSP and HC. The FC of ORB with other regions was found to be associated with neurocognitive and social cognition^54,55^, and the activation of ORB was likely to be found when individuals were in a threat context^56^. The decreased FC between the ORB and the temporal lobe, in VSP may be interpreted as incorrect processing of auditory verbal stimuli. Thus, the patients may have difficulty distinguishing reality from auditory verbal hallucinations, potentially leading to an increased risk of violence influenced by these hallucinations. Our findings are supported by evidence of a close correlation between auditory verbal hallucinations and violence in patients with schizophrenia^57,58^. Even after controlling for positive symptoms in the patients, the VSP still exhibited decreased FC between the MTG and the ORB, suggesting that abnormal processing of hallucination signals may be an underlying mechanism of violence. Although previous functional neuroimaging studies have partially supported the involvement of these regions in aggression, the specific ORB-MTG connectivity identified here has not been established as a core mechanism. Therefore, our findings should be considered exploratory and warrant further investigation in independent samples.

Alterations of the frontal pole are one of the features of VSP compared to NVSP. The present study found decreased FC of ORB with both MFG and SFG in the VSP. Consistent with our study, a previous study also found reduced FC between the frontal pole and other brain regions associated with aggression^59^. The MFG, which is involved in a variety of cognitive processes such as information processing and transfer, has been extensively studied^60^. Decreased FC in those areas might be contributive to abnormal information processing and increased vigilance^61^, resulting in a higher risk of aggression. Furthermore, the correlation between deficits in the SFG and hostility, as indicated in a prior study, further supports our results^62^. In summary, our findings provided further evidence that abnormal FC between the ORB and specific frontotemporal regions might be one of the neurobiological mechanisms of violence in schizophrenia. Through further studies, these connections may serve as biological markers for the intervention of patients with schizophrenia who are at a higher risk of violence.

Prior studies have emphasized dysfunctions in frontal-limbic circuits, particularly OFC–ACC and OFC–amygdala connectivity^63,64^, as neural substrates underlying aggression through impaired impulse control and emotion regulation^65^. However, such differences were not observed in the present study. To match the violent and non-violent schizophrenia groups on positive symptoms, our participants all exhibited prominent hallucinations and delusions, resulting in relatively high positive symptom scores between groups, which may have influence group differences in these inhibitory networks, while highlighting connectivity alterations related to positive symptom processing Instead, our findings focused on altered connectivity between the ORB and temporal-frontal association regions, which related to social cognition, auditory processing, and vigilant attention. These connections may play a more important role in violent behavior among patients with severe positive symptoms.

It has been found that exposure to CT may affect the development and functioning of certain brain regions^66^, thereby influencing the FC between brain regions involved in emotional regulation and cognition. The present study revealed that decreased FC between the ORB and the MTG might accounted for the association between exposure to CT and lifetime violence in adulthood. Numerous studies have shown that CT is associated with the volume and blood oxygenation level-dependent responses in the orbitofrontal cortex^24,67,68^. The ORB is involved in neurocognitive and social cognition, while the MTG plays an important role in auditory verbal hallucinations, and both regions are correlated with positive feelings in life^14^ and might be affected by CT. Therefore, exposure to CT may regulate the perception of hallucinations by altering the FC between the ORB and the MTG, thereby increasing the risk of violence. In line with the present study, another study found the important role of the orbitofrontal cortex in CT and aggression^24^, and the reduced FC between the ORB and other brain regions in relation to violence also supports our findings^59^. Moreover, previous studies have established the relationship between CT and heightened threat anticipation as well as reduced stress resilience^69,70^. Patients with history of emotional abuse might predisposing individuals to hypervigilance in threatening contexts^71^. The impaired integration between ORB and MTG, which are involved in hallucination processing and socio-emotional evaluation, might lead to an overestimation of perceived threat following CT and increase the risk of aggression when faced with provocation. This may represent a neural mechanism linking CT, abnormality FC and violence. Taken together, these interacting factors may contribute to heightened aggression risk among patients with schizophrenia who have experienced childhood trauma, underscoring the need for future interventions that integrate neurobiological, psychological, and social approaches.

There are several limitations to the present study. Firstly, the sample size was relatively small for mediation analysis, which might have limited the reliability of our results. Future studies should replicate these findings in independent cohorts to confirm t20heir robustness and generalizability. Secondly, the level of education was significantly lower among the violent patients; although this factor was used as a covariate, the influence of education might not have been fully estimated. Thirdly, all the participants in the present study were male, which might have introduced gender bias into our results. Finally, although mediation analyses were restricted to CTQ subscales and functional connections with significant group differences, formal multiple comparison correction was not performed across models, which increases the risk of type I error. Future studies with larger samples and pre-registered mediation hypotheses are needed to validate these preliminary findings.

## Conclusion

In summary, this study found differences in the FC network between violent patients with schizophrenia, non-violent patients with schizophrenia, and healthy individuals, suggesting that alterations of FC between certain brain regions may be associated with violence. We also found that childhood trauma might contribute to violent behavior in patients with schizophrenia by affecting the FC between ORB and MTG, which can be a potential neural target for interventions of patients with schizophrenia who have experienced childhood trauma and are at a higher risk of aggression.

## Data Availability

The data supporting the results of this study are available upon request from the corresponding author.
